# Association between mental health workforce supply and clusters of high and low rates of youth suicide: An Australian study using suicide mortality data from 2016 to 2020

**DOI:** 10.1177/00048674231192764

**Published:** 2023-08-22

**Authors:** NTM Hill, H Bouras, LS Too, Y Perry, A Lin, D Weiss

**Affiliations:** 1 Telethon Kids Institute, Nedlands, WA, Australia; 2 Centre for Child Health Research, The University of Western Australia, Crawley, WA, Australia; 3 Melbourne School of Population and Global Health, The University of Melbourne, Parkville, VIC, Australia; 4 Curtin School of Population Health, Curtin University, Bentley, WA, Australia

**Keywords:** Suicide, self-harm, mental health

## Abstract

**Objective:**

To examine the association between mental health workforce supply and spatial clusters of high versus low incidence of youth suicide.

**Methods:**

A cross-sectional analysis of spatial suicide clusters in young Australians (aged 10–25) from 2016 to 2020 was conducted using the scan statistic and suicide data from the National Coronial Information System. Mental health workforce was extracted from the 2020 National Health Workforce Dataset by local government areas. The Geographic Index of Relative Supply was used to estimate low and moderate-to-high mental health workforce supply for clusters characterised by a high and low incidence of suicide (termed suicide hotspots and coldspots, respectively). Univariate and multivariate logistic regression was used to determine the association between suicide clusters and a range of sociodemographic characteristics including mental health workforce supply.

**Results:**

Eight suicide hotspots and two suicide coldspots were identified. The multivariate analysis showed low mental health workforce supply was associated with increased odds of being involved in a suicide hotspot (adjusted odds ratio = 8.29; 95% confidence interval = 5.20–13.60), followed by residential remoteness (adjusted odds ratio = 2.85; 95% confidence interval = 1.68–4.89), and illicit drug consumption (adjusted odds ratio = 1.97; 1.24–3.11). Both coldspot clusters occurred in areas with moderate-to-high mental health workforce supply.

**Conclusion:**

Findings highlight the potential risk and protective roles that mental health workforce supply may play in the spatial distributions of youth suicide clusters. These findings have important implications for the provision of postvention and the prevention of suicide clusters.

## Introduction

Suicide is a critical public health problem. Each year, approximately 800,000 people die by suicide (Naghavi, 2019; World Health Organization [WHO], 2014), accounting for approximately 34.6 million years of life lost (Naghavi, 2019). The risk of suicide increases markedly during middle to late adolescence (Cha et al., 2018) and remains among the top five leading causes of death in people under the age of 25, globally (Naghavi, 2019). In Australia, suicide is the leading cause of preventable deaths in young people aged 10–24 years (Australian Bureau of Statistics [ABS], 2021). Although the year 2021 saw a decrease in Australian youth suicide rates for the first time in the last decade, overall rates of youth suicide remain notably higher than previous years (14.9 per 100,000 in 2021 (ABS, 2021) compared to 10.2 per 100,000 in 2010 (ABS, 2018a).

Spatial statistical techniques which identify areas where the incidence of suicide is highest, also known as suicide clusters, have the potential to identify the emergence of suicide clusters in real time (Benson et al., 2022), and equip decision makers with information to direct suicide prevention resources to areas where they are most needed (Caine, 2020; Lai et al., 2020). Despite multiple studies of suicide clusters in Australia over the past 10 years (Cheung et al., 2012; Qi et al., 2014, 2012; Robinson et al., 2016; Too et al., 2019; Torok et al., 2017), spatial statistics have not been widely used to drive evidence-informed suicide prevention interventions. Studies of suicide clusters in Australia have been largely limited to the examination of broad sociodemographic factors such as sex, remoteness, Aboriginal and/or Torres Strait Islander status and socioeconomic status (Cheung et al., 2012, 2013; Hill et al., 2020; Robinson et al., 2016; Too et al., 2017a). These studies have consistently found that people who reside in remote areas, characterised by low socioeconomic status, and who are Indigenous Australians are more likely to be involved in suicide clusters (Cheung et al., 2012, 2013; Hill et al., 2020; Robinson et al., 2016; Too et al., 2017a). However, the same risk factors are associated with suicide in the general population and although highly sensitive to suicide, lack the specificity to drive population-level suicide prevention interventions in geographic areas where need is greatest.

Literature in Australia and internationally that has used gold standard spatial scan statistics have largely attributed suicide clusters to a phenomenon known suicide contagion (Benson et al., 2022). Suicide contagion describes the occurrence of multiple suicide deaths following certain types of media reports of suicide and/or following the known or suspected suicide of a friend, family member, acquaintance or member of the community (Cheng et al., 2014; Hawton et al., 2020). Previous studies of suicide clusters suggest that young people are particularly susceptible to the effects of suicide contagion (Hawton et al., 2020). However, these studies did not measure whether cluster members were known to one another (Gould, 1990; Gould et al., 1990; Niedzwiedz et al., 2014; Robinson et al., 2016), leading to significant biases in the field (Hill et al., 2020). In contrast, our previous research showed approximately 10% of young people who died in youth suicide clusters, detected using spatial scan statistics (Hill et al., 2020), were known to each other, highlighting that some, but not all suicide clusters detected using this technique may be driven by contagion mechanisms.

The association between suicide clusters and access to healthcare services is an under-researched, yet promising avenue for understanding the association between existing contextual factors associated with suicide clusters. For example, shortages in the mental health workforce, limited opening hours, and poor access to mental health services may deter individuals from seeking or receiving help during a suicidal crisis. The same factors have been shown to be more pronounced in geographically remote and socioeconomically disadvantaged areas and thus may provide a more precise account for existing trends in suicide clusters across different communities. Importantly, these factors are modifiable. In countries such as Denmark and Finland (Erlangsen et al., 2015; Pirkola et al., 2009), service reform resulting in increases in the availability of community mental health services has been linked to an overall reduction in suicide rates across the lifespan, suggesting that population-based strategies associated with access to mental health services may have upstream suicide prevention benefits (Erlangsen et al., 2015; Korosec Jagodic et al., 2013; Pirkola et al., 2009). However, the role of access to healthcare services has yet to be examined in the context of suicide clusters in Australia and internationally.

This study seeks to redress this gap in evidence by examining the association between spatial clusters of suicide characterised by greater than expected rates of suicide and lower than expected rates of suicide (referred to hereafter as suicide hotspots and coldspots, respectively). The use of the spatial scan statistic to determine areas of high versus low cluster events has been widely documented in the environmental sciences (Fritz et al., 2013) and across a range of disease burdens including heart disease (Caswell, 2016; Cui et al., 2019; Liu et al., 2015; Shaikh and Malik, 2019), malaria (Gopal et al., 2019; Tewara et al., 2018), hepatitis (Jiang et al., 2015) and depression (Salinas-Pérez et al., 2012). Identification of spatial hotspots and coldspots of disease and health-related behaviours have had considerable impact across healthcare disciplines, for example, informing the national roll-out of public health campaigns including immunisation (Eccles and Bertazzon, 2015; Figueira et al., 2003; Kamadjeu and Tolentino, 2006; Monguno, 2013; Omer et al., 2008).

## Method

We used the scan statistic to identify suicide hotspots and coldspots and investigated the association between these clusters and mental health workforce supply. The scan statistic method is a statistical approach utilised to detect and assess the significance of clusters within a given data set (Kulldorff, 2018). It involves employing a scanning window, which can be circular or of varying shape, to move across the data space. By comparing observed cluster statistics with expected values under the null hypothesis, the scan statistic determines whether the observed clustering is statistically significant or occurs by chance. This study received approval from the Department of Justice and Community Safety Human Research Ethics Committee (CF/21/2962).

### Suicide estimates

We identified coroner confirmed cases of suicide (ICD-10 codes X60–X84) in young people (aged 10–25 years) and adults (aged 26 and above) who died in Australia between 1 January 2016 and 31 December 2020 using the National Coronial Information System (NCIS). Data from 2021 onwards were excluded as more than 70% of cases remained open at the time of the analysis. The NCIS is a national online database that records all reportable causes of deaths in Australia and New Zealand. The NCIS provides core demographic information, information on the methods and cause of death, toxicology findings and residential address for each case recorded in the database (Saar et al., 2017). Geocoded point estimates for the residential address (longitude and latitude coordinates) were extracted for each case, along with key demographic information recorded by the NCIS core data set including date of suicide incident, time of incident, age, sex, Aboriginal and/or Torres Strait Islander status, employment and education status, method of suicide, remoteness and the use of illicit drugs and psychopharmaceuticals (e.g. antidepressants, antipsychotics, anxiolytics) and alcohol determined by positive toxicology findings.

### Mental health workforce data

We extracted the number and full-time equivalent (FTE, a unit of measurement that represents a 40-hour standard work week) of mental health workers in each local government area (LGA) using publicly available data from the 2020 National Health Workforce Dataset (NHWDS) (Department of Health and Aged Care, 2022). LGAs are legally designated areas for which local governing bodies are responsible and are thus determined to be the most relevant spatial unit for decision makers at a population level. Mental health workers consisted of psychiatrists, mental health nurses, psychologists and mental health occupational therapists. While primary health care clinicians also play important roles in mental health care, we have decided to focus solely on the above-mentioned occupations as they are a specialist pathway for mental health support. Primary health care clinicians’ contributions often only involve initial assessments, referrals and coordinating care. Psychiatrists were extracted from the ‘primary specialty’ tab, mental health nurses from the ‘job area as a nurse’ tab, psychologists from the ‘professions’ tab and mental health occupational therapists from the combination of the ‘scope of practice’ and the ‘professions’ tab. Data recorded in the NHWDS are recorded by the Australian Health Practitioner Regulation Agency (AHPRA) workforce survey in combination with information on annual registrations (Department of Health and Aged Care, 2022). In 2020, the response rate to the NHWDS was >90% (Department of Health and Aged Care, 2022). We imputed missing data by conducting a desktop scan of services in 70 LGAs with missing information or zero counts based on publicly available information from the National Health Services Directory (NHSD) and contacted relevant services for estimates on the number of mental health clinicians and their FTE.

### Area-level socioeconomic status and remoteness

We extracted decile scores from the index of relative socioeconomic disadvantage (IRSD) recorded by the 2020 ABS Census (the most recent publicly available Census statistics) for each LGA (ABS, 2018b). The IRSD is a measure of the economic and social conditions of people and households within an area. Low scores represent greater disadvantage (e.g. low incomes and few skilled occupants), whereas high scores represent the least disadvantage. Disadvantage referred to the top 20th percentile of disadvantage for each LGA (Hill et al., 2020). Remoteness was extracted for each LGA using data from the 2020 ABS Census and included (1) major city, inner and outer regional, and (2) remote and very remote communities (ABS, 2016).

### Travel time

Travel time to mental health services was estimated using established methodology that estimates the number of minutes required to reach a location providing a service using existing transportation infrastructure and typical rates of travel (Weiss et al., 2018, 2020). The service locations consisted of emergency departments open 24 hours per day and ‘headspace’ facilities. Headspace is the largest youth mental health provider in Australia with over 150 centres across the country and provides mental health support to young people aged 12–25. The location and operating hours of headspace facilities were obtained from publicly available information on the headspace website (headspace, 2023) and converted to Australian Eastern Standard Time. Australia-wide travel time maps were generated for each 168 hourly blocks constituting a typical (non-holiday) week for the combined sets of emergency department and headspace facility locations. Finally, the map value for each location in the NCIS data set was extracted from the hourly map aligned with the estimated time of death.

### Mental health workforce supply

Mental health workforce supply was estimated using the Geographically adjusted Index of Relative Supply (GIRS) (Australian Institute of Health and Wellbeing (AIHW), 2016). The GIRS is a measure that takes the known mental health workforce supply in an area and adjusts it for land size, population dispersion and the approximate travel time of each case to the nearest headspace centre or emergency department depending on the time of suicide incident recorded in the NCIS, relative to the opening hours of the nearest headspace centre. If the incident occurred outside operating hours, travel to the nearest emergency department was used.

The GIRS score comprised four components with each component representing a score of 0–2 that was calculated using interquartile ranges. The four components included (1) mental health workforce supply determined by FTE rates of mental health clinicians per 100,000 using 2020 LGA population estimates as the denominator; (2) land size of each LGA measured in square kilometres, extracted from the from the 2020 ABS LGA shapefile; (3) population dispersion determined by dividing the LGA’s estimated resident population by its square kilometres; (4) travel time based on the distance of each case at the time of incident (recorded in the NCIS) to the nearest open headspace centre or 24/7 emergency department. A travel time of less than 15 minutes was considered the best-case scenario and travel times greater than 45 minutes the worst-case scenario. Thus, individuals who died by suicide who lived more than 45 minutes from the nearest headspace centre or emergency department at their time of death were assigned a value of 0 and individuals who lived less than 15 minutes away were assigned a value of 2. For a detailed explanation of the method used for assigning the scores to the four GIRS components, see Table 1.

**Table 1.** Method for assigning scores to the four GIRS components.

Score	Range of values to which score assigned, by GIRS component			
	Mental health FTE rate	Population density	Land size	Travel time
0	Lowest 25% of FTE rates	Least densely populated 25%	Largest 25%	Greater than 45-minute travel time
1	Middle 50% FTE rates	Middle 50%	Middle 50%	Between 15- and 45-minute travel time
2	Highest 25% of FTE rates	Most densely populated 25%	Smallest 25%	Less than 15-minute travel time

GIRS: Geographically adjusted Index of Relative Supply; FTE: full-time equivalent.

To calculate the GIRS score, each of the four components above were combined yielding a score that ranged between 0 and 8 for each suicide case. A GIRS score of 0 indicates areas that are likely to face the most challenges in terms of mental health workforce supply. These areas are large, sparsely populated and ranked among the bottom 25th percentile of mental health FTE. A score of 8 indicates that the person who died by suicide lived in an LGA that is small and densely populated is ranked in the top 25th percentile of mental health FTE and has good access to mental health services. The GIRS score was combined into a categorial variable characterised by low mental health workforce supply (GIRS scores 0–3) and moderate-to-high mental health workforce supply (GIRS scores 4–8).

### Statistical analysis

We used the spatial scan statistic to detect LGA’s suicide hotspots and coldspots using SatScan version 10.0.2 (Kulldorff, 2022). Point estimates for each suicide were aggregated to the centroid of the corresponding LGAs using Arc GIS software. Population estimates from 2020 were extracted for each LGA from the ABS Table Builder. The Poisson discrete scan statistic was used to detect high and low relative rates of suicide incidence for each Australian state and territory, using a circular scanning window. The maximum spatial window was set at 10% of the population at risk (Hill et al., 2020) and the likelihood of each possible cluster was assessed using Monte Carlo simulations. Clusters were included if their *p* value was <0.10 to account for the statistically rare incidence of suicides (Hill et al., 2020; Robinson et al., 2016; Too et al., 2019). Clusters are referred to as ‘possible clusters’ in the 0.10 < *p* > 0.05 range and ‘significant clusters’ if *p* < 0.05 (Hill et al., 2020; Too et al., 2017b, 2017a).

We used descriptive statistics to describe the demographic characteristics of young people who died in suicide hotspots, coldspots and non-cluster suicides. A comparison of demographic characteristics among hotspot, coldspot and non-cluster suicides was conducted using Pearson’s chi-square test of independence. Significant differences were determined by *p* values <0.05.

We used univariate logistic regression to examine differences in sociodemographic characteristics (Aboriginal and/or Torres Strait Islander status, remoteness, Not Enrolled in any Education, Employment, or Training [NEET] status, Index of Social Disadvantage, drug and alcohol use, and mental health workforce supply between suicide hotspots and coldspots, with non-cluster suicides as the reference group). We used multivariate analysis to examine the strength of association between suicide hotspots and low mental health workforce supply controlling for the variables outlined above. The association between mental health workforce supply and suicide coldspots was not conducted due to perfect separation (all areas were characterised by high mental health supply).

All analyses, except for the scan statistic, were conducted using R version 4.2.0.

## Results

### Characteristics of suicide clusters

**Figure 1.**
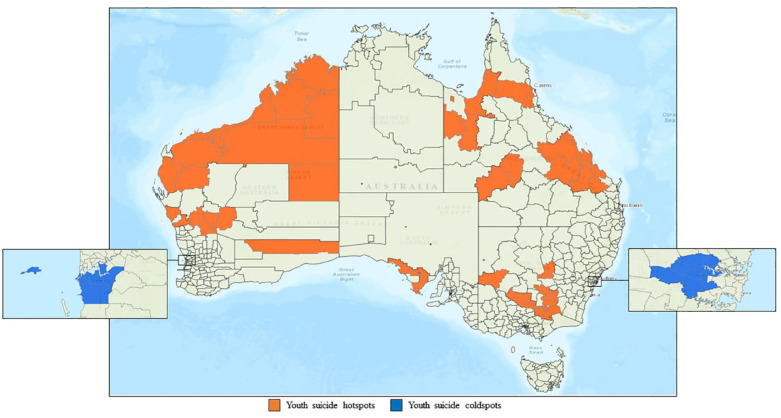
Location of youth suicide hotspots and coldspots in Australia by local government area, 2016–2020.

Between 1 January 2016 and 31 December 2020, eight hotspots (clusters of high relative risk) were detected across six Australian states and territories (Figure 1). Hotspot communities comprised 228 young people, accounting for 12% of youth suicides during the study period (Table 2). The number of mental health workers in hotspot communities was 875 per 100,000, accounting for approximately 739.7 FTE hours per 100,000. For areas with low relative risk of suicide, two coldspot clusters were identified. The two coldspots comprised 87 young people, accounting for 4% of youth suicides. The number of mental health workers in coldspot communities was 1627 per 100,000, accounting for approximately 1346.3 FTE hours per 100,000.

**Table 2.** Overview of the detected high- and low-risk cluster areas using the scan statistic.

Cluster location	Cluster	Cluster	Years	Cluster duration	LGA areas	cluster cases	Total suicides	*p* value
				*N* (years)	*N*	*N* (%)	*N*	
ACT_NSW	Hotspot	1	2016–2020	5	20	40 *(6)*	649	*p* < 0.05
ACT_NSW	Coldspot	1	2016–2020	5	8	64 *(10)*	649	*p* < 0.05
TAS	Hotspot	1	2017, 2019–2020	4	1	8 *(22)*	37	*p* < 0.05
TAS	Coldspot	^a^	^a^	^a^	^a^	^a^	^a^	^a^
SA	Hotspot	1	2016–2019	4	10	8 *(7)*	116	*p* < 0.05
SA	Coldspot	^a^	^a^	^a^	^a^	^a^	^a^	^a^
QLD	Hotspot	1	2016–2020	5	20	30 *(6)*	527	*p* < 0.05
QLD	Hotspot	2	2016–2017, 2019–2020	5	1	5 *(1)*	527	*p* < 0.05
QLD	Hotspot	3	2016–2020	5	9	67 *(13)*	527	*p* < 0.05
QLD	Coldspot	^a^	^a^	^a^	^a^	^a^	^a^	^a^
VIC	Hotspot	1	2016–2020	5	7	21 *(7)*	318	*p* < 0.05
VIC	Coldspot	^a^	^a^	^a^	^a^	^a^	^a^	^a^
WA	Hotspot	1	2016–2020	5	26	49 *(19)*	256	*p* < 0.05
WA	Coldspot	1	2016–2020	5	9	23 *(9)*	256	0.10 < *p* > 0.05

LGA: local government area.

a No clusters detected.

### Individual characteristics

The characteristics of young people who died in suicide hotspots, coldspots and non-cluster locations are shown in Table 3. Young people who died in suicide hotspots were more likely to be Aboriginal or Torres Strait Islander compared to suicides in coldspots and non-cluster locations (27% vs <5% and 10%, respectively; *p* < 0.05; a similar trend was observed for suicides that occurred in remote and very remote locations). Young people not enrolled in any education, employment, or training were more frequently involved in suicide hotspots (52%) compared to coldspots (32%) and non-cluster locations (43%). Rates of illicit substance use were significantly higher in hotspots (25%) compared to non-cluster locations (15%), but no differences in illicit substance use were observed between hotspots and coldspots. A similar trend was observed for alcohol consumption at the time of death. The remaining demographic characteristics of young people who died by suicide were comparable in hotspots, coldspots and non-cluster locations.

**Table 3.** Overview of the demographic characteristics of young people in hotspots, coldspots and non-cluster cases.

Characteristic	Hotspot cases	Coldspot cases	Non-cluster cases	*p* < 0.05
	228 (12%)	87 (4%)	1644 (84%)	Hotspot vs coldspot Hotspot vs non-cluster cases Coldspot vs non-cluster cases
Sex (male)	173 (76%)	67 (77%)	1221 (74%)	–
Aboriginal and/or Torres Strait Islander status	61 (27%)	<5	166 (10%)	Hotspot vs coldspot
Resides in a remote and very remote location	64 (28%)	<5	42 (3%)	Hotspot vs non-cluster cases
Not in employment, education or training	118 (52%)	28 (32%)	704 (43%)	Hotspot vs coldspot Hotspot vs non-cluster cases
Psychotropic medication detected at the time of death	22 (10%)	9 (10%)	130 (8%)	–
Illicit drugs detected at the time of death	58 (25%)	13 (15%)	243 (15%)	Hotspot vs non-cluster cases
Alcohol detected at the time of death	62 (27%)	13 (15%)	290 (18%)	Hotspot vs non-cluster cases
Resides in the 30% most disadvantaged regions	57 (25%)	69 (79%)	440 (27%)	Hotspot vs coldspot

### Association between cluster membership and mental health workforce supply

Tables 4 and 5 show the variables that were associated with suicide hotspots and coldspots, respectively. Results of the multivariate logistic regression show that low mental health service supply was associated with greater odds of being involved in a suicide hotspot (odds ratio [OR] = 8.29, 95% confidence interval [CI] = 5.20–13.60). The investigation of mental health supply in coldspot locations was not conducted due to zero counts (all coldspots were characterised by moderate-to-high mental health workforce supply).

**Table 4.** Association of sociodemographic characteristics and mental health workforce supply and suicide hotspots.

Hotspots	Unadjusted odds ratio	95% CI	*p* value	Adjusted odds ratio	95% CI	*p* value
Low mental health workforce supply	28.4	19.55–42.45	<0.01	8.30	5.20–13.60	<0.01
Sex (male)	1.09	0.75–1.52	0.33	1.18	0.78–1.80	0.43
Aboriginal and/or Torres Strait Islander status	3.25	2.32–4.53	<0.01	1.32	0.80–2.15	0.27
Not in employment, education or training	1.43	2.32–4.53	<0.01	1.06	0.74–1.53	0.73
Residential remoteness	4.89	1.09–1.89	<0.05	2.85	1.68–4.89	<0.01
Psychotropic medication detected at the time of death	0.8	9.81–22.82	<0.01	1.58	0.83–2.94	0.15
Alcohol detected at the time of death	1.74	0.51–1.32	0.37	0.95	0.61–1.46	0.81
Illicit drug detected at the time of death	1.97	1.26–2.39	<0.01	1.97	1.24–3.11	<0.01

OR: odds ratio; CI: confidence interval.

**Table 5.** Association of sociodemographic characteristics and mental health workforce supply and suicide coldspots.

Coldspots	Unadjusted odds ratio	95% CI	*p* value	Adjusted odds ratio	95% CI	*p* value
Low mental health workforce supply						
Sex (male)	1.16	0.71–1.98		1.12	0.68–1.91	0.68
Aboriginal and/or Torres Strait Islander status	0.21	0.034–0.67	<0.05	0.23	0.04–0.74	0.04
Not in employment, education or training	0.63	0.39–0.99	0.052	0.69	0.43–1.10	0.13
Residential remoteness						
Psychotropic medication detected at the time of death						
Alcohol detected at the time of death	0.82	0.43–1.45	0.52	0.85	0.43–1.53	0.60
Illicit drug detected at the time of death	1.01	0.53–1.79	0.98	1.19	0.61–2.19	0.59

OR: odds ratio; CI: confidence interval.

## Discussion

This study examined the association between spatial clusters of suicide and mental health workforce supply in a nationwide study of youth suicide. Specifically, we found areas with low mental health workforce supply were associated with eightfold greater odds of a suicide occurring in a hotspot compared to non-cluster suicides. The association between mental health workforce supply in hotspot communities remained robust after controlling for key sociodemographic covariates including Aboriginal and/or Torres Strait Islander status, remoteness and drug and alcohol use. This evidence is strengthened by our analysis of suicide coldspots (LGAs where the rate of youth suicide were lower than expected), all of which occurred in areas characterised by moderate-to-high mental health workforce supply.

The association between mental health workforce supply and hotspots of youth suicide reported in this study is consistent with hypotheses from previous Coroners’ investigations and descriptive studies of youth suicide clusters in Australia and internationally (Coroners Court of Victoria, 2015; Forbes et al., 2012; Hacker et al., 2008; Kvernmo, 1995; Substance Abuse and Mental Health Services Administration [SAMHSA], 2014). However, unlike previous studies, which used narrative and descriptive methodologies, the current findings were obtained using a rigorous statistical method as well as health workforce and suicide mortality data at a national level.

Previous studies of youth suicide clusters have focused on broad social and demographic characteristics (e.g. sex, remoteness, and socioeconomic advantage and disadvantage) that lack the sensitivity and specificity to inform suicide prevention initiatives at a community level and are therefore not imminently modifiable. In this study, remoteness was associated with threefold greater odds of being involved in a suicide hotspot. The association was strongest, however, in areas characterised by low mental health workforce supply (OR = 8.29). Contrary to previous studies, results of the regression analysis showed that mental health service supply attenuated the association between suicide hotspots and Aboriginal and Torres Strait people (Hill et al., 2020; Qi et al., 2012; Robinson et al., 2016), suggesting that mental health service supply may be a key modifiable risk factor for the prevention of youth suicide clusters.

The finding that hotspots of youth suicide are more likely to occur in areas with low mental health service supply has important implications for the prevention and postvention of youth suicide. Existing community-based postvention and suicide cluster prevention frameworks recommend relevant stakeholders increase screening and referral efforts to mental health and postvention counselling services to avert further suicides and the development of suicide clusters (Palmer et al., 2018; Public Health England, 2019). However, this poses significant challenges in communities with significant mental health workforce shortages (Coroners Court of Victoria, 2015; Hill and Robinson, 2022). A recent evaluation of a youth suicide cluster in Victoria, Australia reported a significant increase in mental health service demand, which led to increased waitlist times and difficulty accessing timely postvention support (headspace, 2016). Another evaluation conducted in Belfast, Ireland noted that existing services were ill-equipped to cope with the demands placed on them resulting in waitlist times to mental health services following a suicide cluster (Forbes et al., 2012). In the latter study, low access to mental health services was associated with frustration and increased distrust in the healthcare service system.

In addition, in communities with significant mental health workforce shortages, strategies that strengthen social support (a known protective factor against suicide) (Abbott and Zakriski, 2014) between community members may be particularly warranted. Findings from this study suggest that postvention and cluster response frameworks ought to be updated to include practical strategies to address increases in mental health service demand in the aftermath of multiple suicides in a community, for example, allocation of emergency funds to extend operating hours and the necessary human capital required to meet increases in mental health referrals (Gardiner et al., 2019, 2020).

As such, policy changes have the potential to further increase the availability of support to those who have the greatest need. A timely solution for communities with existing shortages in the mental health workforce could be the creation of alternative pathways of care through the provision of telehealth services and of low-intensity digital interventions for young people. These types of provision are seen as an effective way to increase accessibility to mental healthcare services and are receiving support from clinicians, young people and parents (Isautier et al., 2020; McQueen et al., 2022).

In this study, young people who showed evidence of illicit substance misuse were twice as likely to be involved in a suicide hotspot compared to those with no known illicit substance use. In Australia, treatment for alcohol and other drug (AOD) use disorders is typically delivered separately to mental health services. A consequence is that mental health services often overlook or exclude AOD disorders, and AOD services have limited capacity to manage people with comorbid mental health concerns. Improved integration of these services has been recognised as a national priority for suicide prevention by The Royal Australian and New Zealand College of Psychiatrists (RANZCP) (2020) and the recent Royal Commission into Victoria’s Mental Health System (State of Victoria). Findings reported in this study further specify improved access to both AOD and mental health services for the prevention of youth suicide clusters.

This study has several limitations. First, the year 2020 was chosen as the reference period as it was the most recent available data at the time of analysis and was therefore considered most relevant to decision makers who may use the study findings. Likewise, data on opening hours and locations of headspace centres used in the analysis of travel time were obtained from 2021 and may have differed somewhat to those that were available during the study period. In addition, we used LGAs as the spatial unit of analysis as these boundaries were considered the most appropriate for informing population-level decision-making on healthcare services. As such, inferences about healthcare service access in smaller geographic regions within LGAs should be conducted with caution (Torok et al., 2019). Finally, this study did not investigate the workforce supply of primary health clinicians who are often the first line of contact, and thus play an integral role for access to mental health services.

## Conclusion

The identification of geographic hotspots of suicide that identify areas where rates of suicide are greater than expected have important implications for suicide prevention, including directing resources to areas where the need is greatest. Findings from this study highlight both the risk and protective role that the mental health workforce supply may play in the spatial distribution of youth suicide clusters. These findings have important implications for the provision of postvention support in the aftermath of a suicide and for the prevention of suicide clusters. Improving access to mental health services can be achieved by increasing the capacity of the mental health workforce, and through the introduction of novel telehealth and digital interventions.
